# Organizational Work, Well-Being, and Quality of Life at an Elderly Age: The Case of Cyprus

**DOI:** 10.1155/jare/7194723

**Published:** 2025-02-19

**Authors:** George Papageorgiou, Panagiota Athanasiadou, Elena Tsappi, Sofia A. Xergia, Athanasios Maimaris, Andreas Efstathiades

**Affiliations:** ^1^Systema Research Center, European University Cyprus, 6 Diogenes St., Engomi, Nicosia, Cyprus; ^2^Department of Physiotherapy, School of Health Rehabilitation Sciences, University of Patras, Rio, Greece

**Keywords:** aging society, elderly people, organizational work, quality of life, retirement, well-being

## Abstract

This study investigates the impact of postretirement organizational work on the well-being and overall quality of life of the elderly population in Cyprus. Specifically, it evaluates the multifaceted effects of continued employment after retirement, based on data collected through a survey administered to a representative sample of elderly Cypriots. The research findings, informed by prominent instruments in the field, demonstrate significant enhancements in autonomy, self-actualization, and both physical and psychological well-being for the elderly as a result of continuing to work beyond retirement age. Through inferential statistical analysis, the study identifies several key factors contributing to significant improvements in people's lives. Particularly, the findings reveal a positive correlation between sustained employment and several aspects of quality of life, challenging conventional views on retirement. These insights hold particular relevance for the Cypriot context and suggest broader implications for policymaking that encourage postretirement employment as a means to enhance well-being in countries with aging populations. Overall, this paper contributes to the discourse on organizational work, aging, and quality of life by offering evidence-based policy recommendations for enhancing human self-actualization and well-being through sustainable professional engagement.

## 1. Introduction

Aging profoundly shapes our social and economic experiences, impacting aspects such as family, work, health, and interpersonal relationships. Life in old age has undergone a significant transformation, especially in the Western world since the mid-20th century. This transformation not only influences individual values and attitudes but also affects self-perception and understanding of life, particularly in the workplace. Consequently, this shift impacts the perception of quality of life (QoL) during aging, a concept deeply intertwined with social, economic, personal, and familial aspects [1, 2].

Demographic shifts, such as decreasing birth rates and increasing life expectancy, coupled with the development of retirement schemes, have reshaped the experience of aging [3]. Earlier research predominantly focused on aging as a stage of incapacity, dependence, and health deterioration [4]. However, since the 1970s, the field of social gerontology has evolved to address the complexities of later life, highlighting the need for research focused on the 55–75 age group [5–7]. This age group, characterized by relative health and economic stability, diverges from traditional roles and contributes significantly to society [7–10]. This shift in perspective has led to the development of multifactorial QoL measurement models, integrating aspects such as health, psychological well-being, morale, autonomy, and physical independence. The modern aging population, benefiting from increased life expectancy, is now more educated, functionally skilled, physically active, and socially engaged than previous generations [11]. This transformation necessitates a comprehensive exploration of the elderly's QoL within social, organizational, and economic contexts [12–14].

Furthermore, the recent COVID-19 pandemic has transformed the way we live by highlighting the role of digital technology in daily life, presenting a significant opportunity to reshape work settings, public health practices, and social behaviors. Studies have shown significant influences on health behaviors, such as adherence to hand hygiene in medical settings, underscoring the pandemic's impact on essential health practices for controlling virus spread [15]. In addition, a recent study emphasizes the integral role of digital technology in fostering resilient built environments, which are crucial for sustainable postpandemic recovery. This highlights how human social capital, when combined with digital innovation, can significantly enhance community resilience, underscoring the importance of these synergies in redefining public health and social practices during global health crises [16]. In particular, digital technology may provide significant support for flexible work scheduling, which would be especially relevant to the elderly population.

In organizational behavior, the productive aging theory suggests that older individuals can continue to make significant contributions to their workplaces and communities. Research indicates that financial independence, autonomy in work decisions, and self-esteem derived from professional roles positively impact the overall happiness and mental health of older adults. This theory supports the idea that postretirement employment can be a crucial factor in enhancing the QoL for the elderly. Social policy perspectives highlight the importance of creating supportive environments that enable elderly individuals to remain active and engaged. Policies that provide flexible work arrangements, combat age discrimination, and promote lifelong learning can significantly improve the QoL of older adults. Recent studies, such as those by Meng et al. and Tabassum et al., have further emphasized the significance of psychological resilience and happiness in educational settings and social interactions, respectively. These studies underscore the profound interplay between mental well-being and social factors in the aging population [17, 18]. Moreover, Mamirkulova et al. explore the role of mega-infrastructure in enhancing QoL in the context of global health crises, presenting a novel perspective on environmental and economic sustainability that affects elderly well-being [19].

The core problem this study aims to address is the lack of clear understanding and empirical evidence on how continued engagement in organizational work postretirement influences the physical and psychological well-being of the elderly population in Cyprus. While some research has highlighted the potential benefits of productive aging, limited exploration exists on how these benefits manifest in specific regional contexts and how policy can be informed by such findings. The identified research gap centers on the need for region-specific studies that examine the multifaceted effects of postretirement employment. Research focusing on the 55–75 age group in Cyprus is scarce, particularly studies that consider the intersection of continued employment and QoL. In addition, existing studies often overlook the cultural, social, and economic nuances that significantly influence the aging experience and the decision to remain in the workforce.

By situating this study within these theoretical frameworks, the research aims to contribute to a deeper understanding of how postretirement work influences the well-being and QoL of the elderly in Cyprus. Building upon the goal of exploring the intricate interrelationships among aging, organizational work, and QoL, this study endeavors to dissect the nuanced impacts of postretirement employment on the well-being of the elderly in Cyprus. Utilizing a comprehensive survey, complemented by both descriptive and inferential statistical analyses, the study seeks to map these dynamics and uncover valuable insights that could inform societal perceptions and policies regarding aging, work, and QoL. We hypothesize that continued engagement in organizational work postretirement significantly enhances the physical well-being of retirees in Cyprus; improves their psychological well-being and mental health; positively influences their self-efficacy, autonomy, and life satisfaction through a balance of employment and leisure activities; and ultimately enhances their overall QoL by providing a renewed sense of purpose, increased social interactions, and financial security.

Central to the investigation are the following pivotal research questions:

• Q1. Extent of influence: How does continued engagement in organizational work postretirement affect the physical and psychological well-being of Cyprus's elderly population? This question aims to explore the depth and scope of work's impact, shedding light on the variance in retirees' health and mental wellness experiences.
• Q2. Balancing act: In what ways do the trade-offs between postretirement employment and leisure activities influence the elderly self-efficacy, autonomy, and life satisfaction in Cyprus? Here, the negotiation between work and leisure is probed, investigating how this equilibrium or its lack thereof shapes their sense of agency, independence, and overall contentment.
• Q3. Role of professional engagement: What role does ongoing professional engagement play in enhancing the elderly's QoL in Cyprus, and through which avenues does it exert its influence? This inquiry seeks to delineate the channels through which continued work enriches retirees' life experiences, considering both direct contributions and subtler, indirect effects.
• Q4. Critical factors: Within the context of postretirement employment, which factors stand out as most instrumental in improving the elderly's QoL in Cyprus? This question directs focus toward pinpointing the aspects of postretirement work, be it social interactions, a renewed sense of purpose, financial security, or the opportunity for skill development, that might be paramount in fostering an enriched life.

Through addressing these research questions, this paper is positioned to analyze the complex interplay between ongoing work engagement postretirement and its repercussions for the elderly's QoL in Cyprus.

## 2. Literature Review

Recent decades have seen a surge in research focusing on QoL, especially since the 1970s, marked by an increase in specialized tools for QoL assessment [20–22]. However, studies specifically examining the QoL of the elderly remain relatively scarce. A search in the EBSCO database illustrates this gap: a general search for “QoL” yields over seven million results but refining the search to include “elderly” narrows it down to just over 27,000. The majority of these studies focus on health-related QoL, as highlighted by works such as in [23–25].

There is an even more pronounced gap in the literature regarding work in the later stages of life and its effect on QoL. While there is ample discussion on the shortage of older workers in the workforce [26, 27], there is limited research on the segment of the aging population that remains active postretirement and how this choice impacts their QoL. Some studies do explore postretirement life, but they seldom focus on the intersection of organizational work and QoL [28–31].

The literature on aging and QoL has expanded to consider the role of technology and online platforms. Abbas, Al-Sulaiti and Al-Sulaiti explore how the pandemic has transformed behaviors, emphasizing the shift towards digital information acquisition and decision-making [32]. This reflects a broader trend where social media and internet use significantly influence public behaviors in constrained environments, such as during a global health crisis.

Moreover, the intersection of technology, innovation, and social behaviors during the pandemic offers new insights into managing public health and social interactions. These changes are crucial for understanding how older adults interact with technology and media to maintain social connections and access health information, which are vital components of their QoL. This research aims to delve deeper into these interrelationships, particularly how postretirement employment and social interactions affect the well-being of the elderly. By incorporating recent findings from studies such as Shuja's, which discusses the psychological impacts of social crises, we can thereby provide a more comprehensive view of the aging experience in today's digitally interconnected world [33].

In addition, the role of psychological well-being and empowerment has been extensively studied in the context of work engagement and employability. Rahi investigated how psychological well-being and psychological empowerment, along with HR practices such as compensation, training, and leadership, significantly influenced work engagement and sustainable employability. This study found that these factors collectively explain a substantial variance in employee work engagement, highlighting the importance of psychological factors in enhancing employee motivation and performance. Moreover, an affective commitment was found to positively moderate the relationship between work engagement and sustainable employability, suggesting that committed employees are more likely to experience long-term employability benefits. These findings underline the need for organizations to focus on psychological well-being and empowerment as key drivers of employee engagement and productivity, which can also be extended to understanding the dynamics of postretirement work engagement and QoL for the elderly [34].

The rather limited studies that do exist on the aging workforce postretirement focus on several key aspects, including financial independence, autonomy, self-esteem, and their correlation with continued organizational work. Financial independence is often linked with improved psychological well-being in the elderly, as they maintain a sense of contribution and purpose. Autonomy in work decisions is found to enhance life satisfaction, giving older adults control over their engagement and schedules.

In addition, self-esteem and a sense of accomplishment, derived from continuing professional roles, positively impact overall happiness and mental health in later life [35–37]. In this context, a recent study provides a systematic review of the psychosocial effects of retirement, shedding light on the complex emotional and social challenges faced by retirees. This work emphasizes the need for a nuanced understanding of the impact of retirement, supporting the study's focus on the multifaceted nature of QoL for the elderly [38].

Furthermore, some studies examined the intersection of work hours, social engagement, and mental health, proposing an extended model of work–life balance for older adults. Findings suggest that moderate work engagement combined with social activities can mitigate depressive symptoms, offering insights into how postretirement work impacts psychological well-being [39]. These factors collectively suggest that organizational work can play a crucial role in enhancing the QoL for the elderly, an area warranting further exploration.

### 2.1. QoL for the Elderly

The QoL for older people is widely reported as a complex concept, as many factors contribute to it. One of the most comprehensive research projects on this topic is the Growing Older (GO) Project, which consists of 24 subprograms. It has led to new insights into the factors determining the QoL of older people and the development of policies to enhance these factors [40]. As a result, new knowledge has emerged on the aging processes of ethnic minorities, the social exclusion of the elderly, the priorities of senior women from different ethnic groups, and the repercussions on the population under investigation [41].

It is important to consider the inequalities in the elderly that are related to their prior socioeconomic conditions. It is consistently confirmed that socioeconomic disparities later in life have a strong correlation with poor health, subfunctionality, and low morale [2, 42–44]. This relates to the concept of “aging well,” which includes three elements: low risk for illness and/or disability, high mental and physical functioning, and active participation in everyday life processes [45]. Several studies highlight the multiple benefits that come from being active at older ages, which could significantly affect QoL. Furthermore, it was shown that such active lifestyles in the elderly can be enhanced by the use of information and communication technology [46–48].

Aging well is directly linked to a variety of psychosocial factors promoted through social inclusion. For example, having good social relationships, a supportive family environment, positive relations with neighbors, living in comfortable homes, and access to the quality public services all contribute to well-being. These social factors directly impact psychological aspects such as optimism, a positive attitude toward others and life, satisfaction, and social acceptance [49, 50]. In addition, the socioeconomic factor of financial security is likely to lead to empowerment and satisfaction, as it allows individuals to maintain independence [51, 52]. Thus, economic independence is closely related to physical well-being and overall prosperity, as it provides individuals with greater control over their lives.

One of the issues consistently raised in surveys on the QoL of older people is the actual context of the social, economic, cultural, and physical environment. This provides a multifaceted experience that essentially concerns the setting in which everyday activities take place. Surveys show that the environment affects how older people perceive their lives. When they evaluate it positively, they often consider factors such as social contact with their family, comparison with the lives of peers in their surroundings, their health, physical conditions, and participation in activities. Conversely, a negative assessment of QoL often emphasizes dependence on others or poor social contacts that lead to misfortune, such as the loss of loved ones [4, 53–56]. A survey study by the authors in [57] confirms the above, emphasizing that it is necessary to include various factors when evaluating the QoL, which go beyond health. However, in another research by the authors in [58], where factors influencing QoL were measured, it was concluded that health is the most widespread response among participants as a factor that could improve or worsen their QoL.

Therefore, health remains a primary determinant in understanding and measuring QoL. Another key factor is participation in social activities and recreation. Participation in social activities is linked to an increasing number of healthy retirement years, where individuals have the opportunity to become active stakeholders in various processes, deriving meaning from their lives through participation in beneficial activities [59, 60]. Creative engagement appears to be a crucial factor influencing how the elderly perceive their environment and thus has a strong correlation with their overall QoL.

Finally, in exploring the dynamics of aging and work, a study proposes a process model that delineates how successful aging at work can be achieved through the interplay between individual motivations and organizational support. This model underscores the importance of adaptability and goal-setting in enhancing job satisfaction and productivity among older workers [61].

What is repeatedly absent from QoL evaluations is the importance of organizational work for the elderly. While concepts such as autonomy, financial security, and material well-being implicitly imply the importance of work, little consideration is given to work beyond retirement. To fill this gap, this paper positions organizational work as a main determinant that could potentially affect the QoL. Furthermore, this study investigates organizational work, aging, and the QoL in the context of Cypriot society, where little is known from previous studies.

### 2.2. Organizational Work and Elderly Life in Cyprus

In Cyprus, the socioeconomic landscape and cultural values could deeply influence the well-being of the elderly. The island's unique history of economic development and cultural heritage shapes its social policies and retirement systems. Specifically, the financial security of retirees in Cyprus could be affected by national pension schemes and private savings, which significantly influence postretirement employment decisions. Social interactions, often characterized by close-knit family structures and community bonds, could play a crucial role in supporting the elderly, thereby impacting their QoL and employment choices postretirement. Moreover, the policy environment in Cyprus, including healthcare and social services for the elderly, may provide a backdrop against which the impacts of postretirement employment can be understood. This setting creates a unique framework for exploring how continued professional engagement affects the elderly autonomy and satisfaction, highlighting the interplay between cultural expectations and individual well-being.

Examining the aging issue from an economic standpoint, significant challenges emerge. Economic thinking aims to create an optimal balance between revenue and expenditure, linked to labor productivity. For profit entities, revenue should consistently exceed costs. However, this balance is disrupted by the aging population. With increasing demands for pensions and health plans, welfare states face higher costs but lower income due to the reduced workforce, leading to crises and consequent cuts in pensions, healthcare, etc. [62, 63]. A plausible solution to the aging problem suggests increasing older people's participation in the workforce, while offering them greater flexibility, such as different kinds of chores, suitable working hours, and shorter working hours [64–66].

For instance, Marchand and Smeeding's economic analysis of retirement indicates that while choosing to stop working immediately after retirement can theoretically increase free time and reduce work, but it may also lead to missed opportunities for additional investment income, such as earning a pension beyond the regular monthly salary. Essentially, this is akin to opting for a riskier investment over a safer one. In addition, retirement equates to zero wage income. This situation could be mitigated by maintaining interest in work among older individuals, through incentives or special arrangements tailored to their needs. Consequently, this may lead to a greater willingness to combine work with retirement [66].

The school of thought, devoted to [67], introduced the term “productive aging.” This term essentially raises the question of whether people aged 65 or over are economically redundant or unnecessary, or if, on the contrary, they represent a vital unexploited source of income that could enable economic growth and/or enhance the welfare of both the state and the people themselves.

Productive aging is an approach that emphasizes the positive aspects of aging and suggests ways in which individuals can make significant contributions to their own lives, their communities, their organizations, and society in general. Productive aging in the workplace calls for a healthy and safe working environment through integrated strategies that enable people of any age group to perform at their best [45, 67].

In addition to innovative approaches to retirement and workforce participation, the dialog on productive aging and the enhancement of elderly individuals' QoL in the workplace has been further enriched by recent research. Wilckens et al. (2021) significantly contribute to this discourse through the introduction of the later life workplace index. This pivotal index serves as a comprehensive tool for evaluating workplace practices, specifically designed to bolster the engagement and productivity of older employees. It underscores the imperative role of the organizational environment in fostering successful aging at work by ensuring that workplace adaptations cater to the needs of an aging workforce, thereby supporting their continued contribution to the economic and social spheres [68].

The concept of productive aging raises the question of whether retirement is ultimately a choice or a social expectation. This is supported in economic terms and raises the question of whether states can provide financial support for 20 years to healthy, well-meaning individuals who are freed from economic productivity [5]. For this reason, it is argued that a free market should exploit previously untapped human resources in cases where there is high demand for selected goods and/or services, and the supply and production require corresponding human resources. This human power, in periods of high demand and low unemployment, could include less traditional workers, such as the elderly/retired [64, 69].

The concept of productive work has been supported by several research studies. In 1991, an American survey conducted by Lewis Harris and associates with a sample of 2999 people aged 55 and above revealed that these individuals were significantly involved in productive activities and wished to continue such involvement when opportunities existed. The most important finding was the unexpected interest among retirees in returning to the labor market [70]. In addition, the research by the authors in [45] provided a detailed discussion of the myths surrounding older citizens, highlighting the need for more flexibility in the labor market to allow seniors to remain active later in life. Finally, various surveys conclude that senior citizens are expected to work beyond statutory retirement ages and to retire gradually, not only because they need additional income beyond their pensions but also because they desire to stay active [3].

In addition to the concepts of productive aging and work, the international literature urgently raises the question of why retirees choose to work or not. To what extent do older people continue to work due to economic circumstances, such as unexpected financial difficulties, and to what extent do they work simply because they enjoy it? This highlights the importance of understanding, both for the state and other stakeholders, the conditions under which older people continue to work in order to create appropriate private and public policies that encourage an increase in labor supply [71–73]. Furthermore, it is important to note that the employment of older people does not uniformly follow the same patterns across different contexts and individuals.

Therefore, the factors that affect the decision to work after retirement are the incentives offered by the various pension schemes, the availability of less physically demanding jobs, the flexibility of the labor market and working hours, and, lastly, the health constraints [30, 74]. For example, in countries such as Japan [75, 76] and the Scandinavian region, they have successfully formally implemented not only gradual retirement plans but also older education programs for older workers to improve their skills and to stay for a long time as a part of the labor market [77–79].

It is also important to see who actually in this age group continues to work. The literature proves empirically that older people who choose to continue to work after retirement are basically financially independent and well-educated [80–82]. On the other hand, those who choose to stop working are mainly employed in areas with higher physical strength requirements, since such work environments usually affect the overall health of individuals [83–86].

Therefore, it becomes clear that there is a need to look deeper into the topic of Aging and organizational work and investigate the potential interrelationships with QoL. Especially, this should be performed within a variety of contexts such as at the country level. In this way, a comprehensive understanding of the possible positive and negative repercussions of working beyond retirement will be gained.

## 3. Methodology

Prior to the full quantitative survey study, the research team conducted a preliminary data collection phase through personal interviews and focus groups. This approach aimed to explore the field, enrich the study with qualitative data, and offer deeper insights into the lived experiences and perspectives of the elderly population engaged in postretirement work in Cyprus. By organizing interviews with a selected group of participants from the targeted elderly demographic, the researchers were able to explore complex themes and nuances that may not be fully captured through survey questions alone. Similarly, focus groups provided a platform for dynamic discussions, revealing shared experiences and diverse viewpoints on the impact of postretirement employment on QoL. This initial stage played a crucial role in fine-tuning the final questionnaire design, ensuring that it comprehensively addressed the relevant dimensions of aging, work, and QoL.

The research follows a pragmatism paradigm, which is appropriate for studies involving both qualitative and quantitative methods. Pragmatism emphasizes practical solutions to research problems and the use of multiple methods to gather data, ensuring a comprehensive understanding of the research problem. This approach is particularly suitable for exploring the complex interactions between postretirement employment and QoL among the elderly in Cyprus, allowing the integration of both qualitative insights from interviews and focus groups and quantitative data from surveys [87].

The choice of mixed methods for this research was guided by the comprehensive approaches in existing studies such as those by Schumacher et al. and Steinmetz et al., which utilize a combination of quantitative and qualitative data to understand the broad demographic trends and specific regional contexts [88, 89]. This methodological framework is crucial for capturing the multifaceted aspects of elderly well-being and postretirement work scenarios.

Acknowledging the intricate relationships between work in later life stages and QoL highlighted in the literature review, this study was designed to explore these relationships within the context of the elderly in Cyprus. A detailed questionnaire was developed to collect data on the actual views of the elderly, focusing on aging, QoL, and organizational work. The construction of the questionnaire was informed by the initial qualitative study analysis and by foundational studies such as WHOQOL-OLD, CASP-19, OPQOL, and WRQoL, ensuring comprehensive coverage of the relevant dimensions. The design of the questionnaire was based on the following studies:

• WHOQOL-OLD [90], produced by the World Health Organization to measure the QoL in the elderly in intercultural contexts.
• Control–Autonomy–Satisfaction–Pleasure-19 items (CASP-19) compiled by the authors in [91] to measure psychological well-being based on QoL.
• Older People's QoL Questionnaire (OPQOL), written by the Health Announcing Professor Ann Bowling, measures the QoL of elderly people [92].
• Work-Related QoL Scale (WRQoL), compiled by a team of British National Health System researchers, measures the QoL and work [93] based on 23 psychometric factors.

The resulting survey instrument comprised 31 questions, divided into seven categories, aimed at measuring the QoL of elderly individuals in relation to their work status. Responses were captured using a Likert scale ranging from 1 (*strongly disagree*) to 5 (*strongly agree*). The questionnaire was administered to a representative sample of elderly individuals in Cyprus, encompassing 121 respondents between 65 and 79 years of age. This sample size was strategically chosen based on a power analysis which calculated the minimum number of responses required to detect a significant effect with 80.

Data collection proceeded with utmost respect for confidentiality, adhering to strict ethical guidelines to safeguard participant anonymity. The survey achieved a response rate of 76%. For the statistical analysis, both descriptive and inferential statistics were employed, including factor and regression analyses. A *p* value of less than 0.05 was considered statistically significant, with all statistical procedures performed using the IBM SPSS software.

For the analysis of survey data, both descriptive and inferential statistical techniques were employed. Descriptive statistics provided a basic summary of the data, while inferential statistics, including factor analysis and regression analysis, were crucial in understanding the relationships between postretirement employment and QoL. Factor analysis was used to identify underlying dimensions within the complex dataset, helping to simplify the data into significant factors related to the QoL. This method is particularly suitable for our study as it allows for the examination of patterns in responses that could indicate broader trends among the elderly regarding their postretirement activities and their impacts. Regression analysis was then utilized to explore the causal relationships between continued employment and various QoL outcomes. This technique was chosen because it is optimal for examining how independent variables (e.g., work engagement) predict or affect dependent variables (e.g., aspects of QoL), providing a clear picture of how postretirement work influences elderly well-being.

The methodology employed to ascertain sample adequacy as exploratory factor analysis was grounded on two pivotal statistical tests: the Kaiser–Meyer–Olkin (KMO) measure of sampling adequacy and Bartlett's test of sphericity. The KMO metric, with values surpassing 0.5 indicating satisfactory sample adequacy, yielded a robust value of 0.801 for this study, affirming the sufficiency of the sample for the analysis undertaken. Concurrently, Bartlett's test reinforced this sufficiency, producing a *ρ* value of less than 0.001, which unequivocally signifies substantial correlations among the variables under investigation, which is in line with the established statistical criteria [94]. The analytical methodology centered around principal component analysis (PCA) for factor extraction, employing varimax rotation to optimize the interpretability of the factor structure. Given the non-normal distribution of scores, the Spearman nonparametric test was judiciously selected for examining variable correlations, while the Kolmogorov–Smirnov test was employed to assess normality. The integrity and internal consistency of the identified factors were rigorously evaluated using Cronbach's alpha, ensuring their reliability. Subsequently, the investigation delved into the influence of postretirement work status on QoL dimensions through meticulous regression analysis.

In crafting a robust and comprehensive understanding of the phenomena at hand, the sampling strategy was meticulously formulated to encompass a wide and representative cross-section of the elderly population in Cyprus engaged in postretirement work. Employing a stratified random sampling method, the study aimed to encompass a broad spectrum of sectors, capturing both formal and informal employment scenarios. Selection criteria were carefully established to include individuals aged 65 years and above, actively involved in some form of organizational work postretirement, with a history of at least 10 years of prior employment. A concerted effort was made to achieve a balanced representation of genders and a diversity of socioeconomic statuses among participants. To this end, recruitment efforts spanned multiple regions within Cyprus, leveraging the networks of local community centers, pensioner associations, and social media platforms to ensure wide and inclusive participant outreach. This deliberate and strategic approach was instrumental in capturing the varied experiences and the nuanced impact of postretirement work on the QoL among the elderly, thereby facilitating a comprehensive and insightful exploration of this significant phenomenon.

In this study, the rigorous selection of statistical methods was paramount to effectively address the research questions concerning the intricate relationships between postretirement work and QoL among the elderly in Cyprus. Utilizing the IBM SPSS software package facilitated the meticulous execution of these procedures, underscoring the study's commitment to methodological excellence. The choice of the KMO measure of sampling adequacy and Bartlett's test of sphericity was instrumental in validating the sample's adequacy for factor analysis. This validation ensured that the dataset was appropriate for the sophisticated analytical techniques applied. The analysis yielded a KMO value of 0.801, indicating a well- suited sample for factor analysis, while Bartlett's test of sphericity confirmed significant intercorrelations among variables with a *p* value of *p* < 0.001.

PCA served as the foundation for factor extraction, with varimax rotation applied to enhance the clarity and interpretability of factor loadings. This approach was particularly salient for uncovering latent dimensions of QoL that may be influenced by postretirement work, aligning closely with the study's objective to explore the multidimensional impacts of continued employment on elderly well-being. In addition, the Spearman nonparametric test was employed for the correlation analysis of variables, a choice dictated by the non-normal distribution of the data. This nonparametric approach allowed for the examination of relationships between variables without presuming a normal distribution. The normality of distribution was assessed using the Kolmogorov–Smirnov criterion, ensuring the robustness of the analytical framework.

The integrity and credibility of the factors identified were further evaluated using Cronbach's alpha, a measure of internal consistency. This step underscored the reliability of the factors derived from the analysis. Lastly, the study explored the impact of working status postretirement on QoL actors through regression analysis, providing a comprehensive understanding of how postretirement employment influences the well-being of Cyprus's elderly population. The detailed exposition of the statistical methods underscores the depth of the analytical approach undertaken in this study.

In the data collection process, careful attention was given to ethical considerations, particularly due to the vulnerability of the elderly population. Prior to data collection, the research protocol was reviewed and approved by an institutional review board (IRB) to ensure that all procedures adhered to ethical guidelines for research involving human participants. Informed consent was obtained from all participants, who were fully briefed on the study's aims, the confidentiality of their responses, and their right to withdraw at any time without penalty. Special care was taken to ensure that the questionnaire and interview processes did not place undue emotional or cognitive strain on participants. Privacy and data protection measures were strictly implemented, with all participant data anonymized and securely stored. These steps underscored the research team's commitment to upholding the highest ethical standards, ensuring the dignity, rights, and well-being of the elderly participants throughout the study.

### 3.1. Data Collection and Variable Selection

The variables selected for this study were meticulously chosen based on an extensive literature review concerning aging, work, and QoL. These variables, namely, physical well-being, psychological well-being, social interactions, financial independence, and autonomy, are central to understanding the dynamics of postretirement employment and its impacts. The selection of variables is justified by their significance in exploring the impact of postretirement work on various aspects of life, as supported by the studies [89, 95, 96].

• Physical well-being: Influenced by global studies on aging such as those by Schumacher et al. [95], this variable addresses the health trajectories and life expectancy crucial for assessing the elderly's living standards. Our study extends these demographics to explore specific health outcomes associated with continuous work beyond retirement, paralleling comprehensive demographic analyses provided by seminal works.
• Psychological well-being: Echoing the findings of Steinmetz et al. [89], who explore neurological burdens, our study includes psychological wellness to gauge mental health's role in enhancing life quality postretirement. This inclusion is critical, considering the significant mental health impacts highlighted in the broader aging research.
• Social interactions: Al-Sulaiti et al. [96] discuss the socioeconomic influences on service evaluations, shedding light on how economic and cultural factors intersect with social interactions. Our research incorporates this variable to understand the social fabric's role in the elderly's life quality, reflecting on how continued work can sustain or even expand these interactions.
• Financial independence and autonomy: These variables address the economic aspects and decision-making freedom that significantly affect elderly life satisfaction. Supported by findings that correlate financial independence with improved psychological well-being and autonomy with enhanced life satisfaction, these variables are crucial for assessing how postretirement work impacts overall autonomy and financial stability.

Each variable was operationalized using validated instruments from prior research, ensuring the reliability and validity of our approach. This methodological rigor substantiates the study's design and reinforces the scholarly contribution, situating our findings within the existing research narrative on aging, work, and QoL. By meticulously linking these variables to documented studies, we provide a robust framework that underpins our exploration of how professional engagement postretirement influences elderly well-being across multiple dimensions.

This study contributes to the existing literature by expanding the understanding of QoL for the elderly. It provides empirical evidence on how continued employment postretirement influences various dimensions of QoL, including autonomy, physical well-being, and psychological health. In addition, the findings offer valuable insights for policymakers in Cyprus and similar contexts, suggesting that encouraging postretirement employment can enhance the well-being of the elderly population. Methodologically, the study employs a mixed-methods approach under a pragmatism paradigm, offering a comprehensive framework for investigating the multifaceted impacts of postretirement employment by combining qualitative and quantitative data for a richer analysis.

## 4. Results

This section presents the main findings of the research, highlighting some of the most significant results. Although the included tables provide comprehensive information, we further provide selective commentary on the findings, focusing on the central research question: Do elderly individuals in Cyprus who remain in the workforce beyond retirement age experience a better QoL compared to those who withdraw from the labor market upon retirement?

As explained in the methodology section, data were collected from a representative sample of 121 elderly individuals.  Table 1 illustrates the demographic characteristics of this sample, including frequency counts and percentages (%) for gender, education level, and work situation, as well as the mean age and its standard deviation.

**Figure 1:**
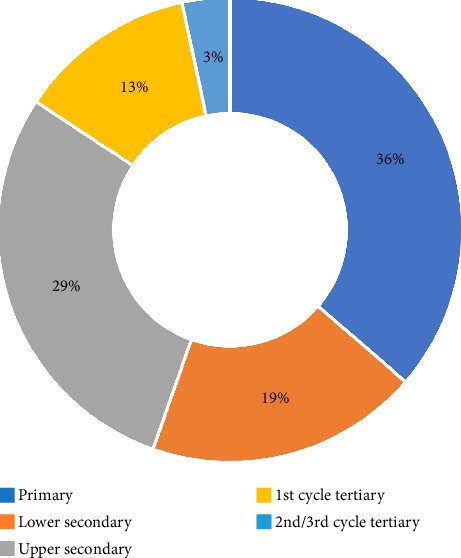
Sample distribution regarding educational level.

Regarding the educational level of the sample, the highest percentage, 36.4% has finished primary school, indicating that these individuals were mainly employed or have been employed in “blue-collar” work. The next highest rate, 28.9%, describes people who have completed high school (i.e., all six upper secondary classes), suggesting that they are mostly white-collar workers. In addition, 19% of the sample, having finished the first three grades of high school, likely falls between these two classes of work.  Figure 1 shows the good representation of the selected sample regarding education level.

Regarding work status, the majority of the sample, 66.9%, declare they do not work after retirement, while 33.1% continue to work after retirement. This aligns with previously presented statistics, noting an increase in the expected healthy years for individuals aged 65 and over in Cyprus, as well as an increase in the working-age population. However, their actual representation within the workforce is declining. Among the four factors presented, gender, age, educational attainment, and work situation, working status (working after retirement/not working after retirement) is the sole independent variable considered, aiming to assess its impact on the dependent variables that constitute the QoL.

As mentioned in the methodology section, the questionnaire comprised 31 statements that delineate the QoL into seven categories. In Table 2, each statement is individually numbered in ascending order. More specifically, the first category, “life in total,” consists of Statements 1–4; the second category, “health status,” comprises Statements 5–8; the third category, “social inclusion,” includes Statements 9–12; the fourth category, “Independence and control,” encompasses Statements 13–16; the fifth category, “emotional well-being,” covers Statements 17–22; the sixth category, “economic aspects,” involves Statements 23–26; and finally, the seventh category, “leisure and activities,” consists of Statements 27–31.

Table 2 displays the sample mean answers and the standard deviations for each statement, analyzed for the entire sample and then segmented by work status after retirement. An independent samples *t*-test was conducted to identify significant differences between the two groups' averages [97]. The *t*-test assesses whether the mean values from two sets of data differ significantly, with a focus on identifying the most notable differences using as a criterion the *p* value (*p* < 0.05) across the seven categories of QoL: Null hypothesis (H0): Work status (working/not working) does not impact the QoL for retirees. Alternative hypothesis (Ha): Work status (working/not working) significantly influences the QoL for retirees.

**Figure 2:**
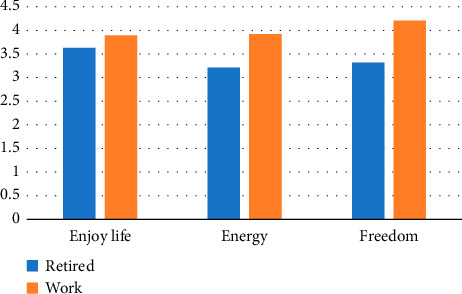
Notable differences in aspects of quality of life with respect to work status.

A low *p* value provides significant evidence supporting the Ha, indicating that work status significantly influences the retirees' QoL. This suggests that retirees who continue to work experience different QoL outcomes compared to those who do not. Conversely, a high p value suggests that work status may not have a substantial impact on the retirees' QoL.  Figure 2 shows some selected significant differences between the elderly who are fully retired and those who continue to work. Notably, freedom and energy levels are significantly higher for the elderly who continue work.

In the category concerning general life aspects, most statements reinforce the H0, suggesting that work status does not significantly influence the general outlook on life for the elderly in Cyprus. Specifically, Item 2, “I'm looking forward to the things that come, now that I retired,” with a *p* value of *p*=0.482, demonstrates that whether an individual is working postretirement does not markedly impact their anticipation for future events. Conversely, Statement 4, showing a *p* value of *p*=0.037, indicates a significant relationship between work status and how retirees cope with challenging life moments. This suggests that senior citizens who continue to work after retirement tend to view life more optimistically, aligning with the Ha [98].

In the “health” category, a particularly noteworthy finding emerges from Statement 5, “I have a lot of physical energy,” where a very low *p* value (*p*=0.001) signifies that the work status of elderly individuals notably impacts their QoL. Continuing to work appears to provide retirees with increased vitality.

However, in the “social inclusion” category, the analysis of social relationships, particularly evident in Statements 9 and 12 with p values of 0.776 and 0.546, respectively, shows that the employment status of the elderly does not significantly affect their interactions with children, friends, and relatives. They report feeling supported regardless of their work status. Therefore, the social connections of Cypriot pensioners appear to remain robust irrespective of their engagement in postretirement work.

In the “independence and control” category, the differences observed are particularly striking. Statements 13 (“I have more independence if I continue to work”), 15 (“I have more control over my future if I continue to work”), and 16 (“I have more freedom to make my own decisions if I work”) reflect on the aspects of independence, freedom, and control among the elderly. The remarkably low *p* value of *p*=0.001 across these statements, coupled with the overall mean, decisively suggests that work status significantly influences the QoL for the elderly. This finding leads to the conclusion that extending one's working life postretirement substantially contributes to greater independence, enhanced control over one's future, and increased freedom in decision-making, as reflected by the elevated mean values for these statements.

Furthermore, the “emotional well-being” category presents intriguing findings. Statements 17 (“I feel more satisfied if I work”) and 19 (“I feel more productive if I work”), both with a *p* value of *p*=0.001, indicate that continuing to work positively impacts the QoL, particularly in terms of psychological health. It is evident that maintaining employment beyond the age of 65 significantly enhances self-esteem and self-confidence among retirees.

In the sixth category, “economic aspects,” I have more freedom to the findings predominantly indicate that the working status of pensioners in Cyprus has a minimal effect on their economic QoL. An exception is noted in Statement 26, “I have the financial capacity to do all the things I enjoy,” where *p*=0.02, challenging the H0 in favor of the alternative. This suggests that, notwithstanding the general economic independence experienced by retirees, those who remain employed postretirement tend to be more financially prosperous.

The final category, “leisure time and activities,” presents intriguing insights. Statements 29, “To continue working gives purpose to my life,” and 30, “I am busier if I continue to work,” with *p*=0.008 and *p*=0.004, respectively, demonstrate that sustained employment has a favorable influence on mental health. This is attributed to the sense of purpose and busyness that work provides, underscoring the positive psychological impacts of continued professional engagement in retirement.

In summary, the analysis of sample means reveals that pensioners who opt to continue working postretirement report a perceptibly higher QoL, characterized by enhanced vitality, independence, and freedom. This indicates a significant psychological benefit, as continued employment is associated with increased life satisfaction. Interestingly, the decision to work or retire does not significantly impact the QoL in terms of social and family relationships. This finding aligns with the cultural context of Cyprus, where the nuclear family plays a pivotal role in providing social support, irrespective of an individual's employment status postretirement. Moreover, while work status does not markedly affect pensioners' perceptions of financial self-sufficiency, a reflection of a generally stable economic status among retirees, those who remain in the workforce do enjoy the added financial advantage, enabling them to afford more luxuries and activities.

### 4.1. Factor Analysis

Furthermore to the above, factor analysis was conducted on the 31 statements of the questionnaire, narrowing down to 19 statements via dimension reduction. These are categorized into five factors: autonomy and life satisfaction, economic independence, physical well-being, social inclusion, and leisure. The factor analysis investigated the structure (factors) among the questionnaire items and explored their relationships [97, 98]. The extracted structure was then used to produce scores for each factor.

As previously mentioned, PCA was carried out to derive the overall variance explained by the fewest possible factors. The number of factors, determined by eigenvalues greater than 1, led us to retain five factors explaining 70%–80% of the total variance. The total variance explained by the extracted factors was 71%, making this a quite credible model.

The item correlations with each factor are presented as “loadings,” as shown in Table 3. The questionnaire items most closely related to the factors informed their naming. Factor scores were derived from the average responses to the questionnaire statements. Factor loadings reveal the relative contribution of each item to its respective factor. For instance, the first factor “autonomy and self-actualization” comprises eight statements, each with significant loadings (greater than 0.3).

Grouping these statements under a single factor provides key insights into their relationship with work, as discussed in the following. The second factor, “financial independence,” consists of 4 significantly loading statements; the third factor, “physical ability,” from 3 highly loading components; and finally, the fourth, “social relations,” and the fifth factor, “free time,” consist of 2 components that are significantly loading the factors.

Also, high rates of explanation of the dispersion of statements are indicators of good adaptation. For example, in the first factor, the dispersion of the first statement (“I have more freedom to make my own decisions if I work”) is interpreted by 76.6% of the dispersion of the model. Accordingly, the high percentages of the eight statements of the first category in combination with the low p values they have, as shown in Table 2, show that the model is well adapted and more specifically, the factor “autonomy and ethical satisfaction” is statistically important in our affairs.

What follows from the factorial analysis is the development of multiple cause-and-effect models via linear regression analysis. In this model, we could investigate the weight of the influence of the independent variable (working status) on the dependent (Model 5's factors) variable. As shown in Table 4, the regression analysis reveals that autonomy, self-actualization, and physical well-being are significantly influenced by continued work beyond retirement, underscoring the positive association between these factors and the decision to remain professionally active.

In examining the dependent variable autonomy and self-actualization, the study observed that the beta (*β*)-coefficient for the independent variable “work” is substantial at 0.669. This indicates a significant positive difference in autonomy and ethical satisfaction between pensioners who are working and those who are not, with a marked increase of 0.69 points for those engaged in labor postretirement.

The effect of the work is also confirmed by the F-test, yielding *ρ*=0.001 (where *p*=*sig*. < 0.05), suggesting that the large *F*-value of 17.840 (indicating robust regression) in combination with the low *p* value demonstrates the independent variable “work” as statistically significant for enhancing the sense of autonomy and moral satisfaction among pensioners. Similarly, the dependent variable “body wellness” is affected in the same way, since the *β*-coefficient indicates that pensioners who continue to work postretirement exhibit a 0.5 points increase in “body wellness” compared to those who do not work.

The statistical significance of the independent variable “Work” in influencing the “physical ability” factor is underscored by a *p* value of 0.002 and a high *F*-value of 9.6958, indicating a robust regression model with a significance level of less than 0.05. These analyses suggest that extending working life beyond retirement positively affects not only the psychological well-being but also the physical well-being of the elderly. This implies that remaining active through postretirement work contributes significantly to both psychological and physical health.

Conversely, the dependent variable “financial independence” does not show statistical significance, as indicated by a relatively low *F*-value of 2.436 and a high *p* value of 0.121. This suggests that continued employment postretirement in Cyprus does not significantly impact retirees' economic situation. This finding aligns with previously mentioned *t*-test results, suggesting that pension income in Cyprus generally suffices for basic living needs but does not support substantial discretionary spending, attributable to Cyprus's relatively high standard of living and the low risk of poverty relative to the poverty line.

Similarly, the dependent variable “socialization” shows no significant impact from the independent variable “Work,” as indicated by an *F*-value of 2.468 and a *p* value of 0.119. This finding suggests that retirees maintain good social relationships with family and friends, regardless of their work status. The absence of a significant difference in human contact between working and nonworking retirees could be attributed to Cyprus's strong emphasis on family values and the high importance placed on maintaining good interpersonal relations. Thus, ceasing work upon retirement does not necessarily lead to a reduction in social contacts established through the workplace.

In examining the five dependent variables, it is observed that the adjusted *R*^2^ (adjusted R-squared) determination coefficient, which represents the proportion of variance explained by the independent variable in the dependent variables, corrected for the sample size, indicates a relatively low percentage of variance explained. The highest values are observed in “independence and moral satisfaction” (12.3%) and “physical well-being” (6.9%), underscoring their relevance to the research question. The modest percentages in this indicator may suggest that the results cannot be generalized for the entire population category under study, potentially due to the limited sample size. Another interpretation could be that this specific independent variable alone may not sufficiently explain the variations in the dependent variables, indicating the necessity for additional factors not measured in this study. However, the focus was on the impact of “work” on aspects of QoL.

In summary, the regression models indicate that retirees who continue working postretirement tend to experience higher levels of autonomy, moral satisfaction, and physical well-being compared to those who cease working upon reaching retirement age. In addition, it is noteworthy that retirees' financial capacities generally meet their living needs and obligations, and they maintain robust social relations with their family and social circles, regardless of their employment status.

A fundamental aspect of this regression analysis was the examination of normality, conducted using the Kolmogorov–Smirnov criterion. The results indicated that the data did not follow a normal distribution. The credibility of the factors was assessed using Cronbach's alpha internal consistency criterion.  Table 5 presents both the normality test results and the reliability measures for the factors.

From the analysis above, Cronbach's alpha internal consistency index, which measures the credibility of the factors, shows that the first three factors appear reliable, as values over 0.6 are considered acceptable. The lower values in the last two factors (“social relations” and “leisure time”) could be explained by the small number of items that load well on the factors.

Since the normality criterion was not met in the dataset it was deemed necessary to analyze the correlations between the factors according to the nonparametric coefficient *ρ* of Spearman to check whether they were altered simultaneously or not. If the correlation is positive, then the two variables tend to change in the same direction, that is to increase or decrease at the same time. If the correlation is negative, then the two variables move in opposite directions, that is, when they grow, one decreases. The closer the values are to −1 and +1, the greater is the relationship that links the variables [98].  Table 6 shows the correlations of the five factors by Spearman's *ρ*-factor. Let us comment on the main findings.

Between physical well-being and financial independence, the correlation coefficient (*ρ*=0.352^∗∗^) is low, positive, and statistically significant at a significance level of 0.01. Therefore, pensioners with more financial independence tend to have relatively more energy since they remain active. Similarly, between physical well-being and leisure time, the correlation coefficient (*ρ*=0.354^∗∗^) is low, positive, and statistically significant at a significance level of 0.01. Thus, pensioners who fill their time both qualitatively and quantitatively with meaningful activities tend to have relatively more physical energy.

Furthermore, there is a moderate positive relationship between leisure time and financial independence (*ρ*=0.209), with a significance level of *p* < 0.05, suggesting that retirees who engage in more activities tend to have relatively more financial independence. The correlation coefficients among the other factors are nil and statistically insignificant, indicating that no reliable conclusions can be drawn about the relationships between them.

As shown by the analysis of the averages of the statements included in the positively correlated factors, retired people who continue to work tend to be more financially independent and are able to spend more than those who do not work. In addition, elderly people who continue to work tend to score higher in terms of physical energy, well-being, and engagement in active and/or new activities, compared to those who retire from work. Thus, greater financial independence and physical well-being among these working retirees provide them with more opportunities to engage in desired activities and continuously explore new hobbies and interests. In conclusion, through the above analysis, the following conclusions can be drawn, which will be further analyzed in the next chapter of the results' discussion:

• The *t*-tests made for all statements make it clear that the physical well-being, autonomy, and self-actualization of people who choose to continue to work is on average higher in relation to those who stop working.
• Through the factorial analysis and then the simple linear regression models, it was shown that the five factors identified as dependent variables with respect to the independent variable “work” are statistically significant for the components that examine physical energy and psychological well-being in older people. However, for the factors dealing with financial condition and social relationships, work after retirement does not show statistical significance, for reasons explained earlier.
• Finally, through the correlations made between the model of five factors, there appear to be positive correlations between financial independence, physical well-being, and leisure time.

## 5. Discussion

This study has investigated the nuanced effects of postretirement organizational work on the QoL among the elderly in Cyprus, providing insights into the complex interplay between continued professional engagement and well-being in later life. Our findings highlight significant improvements in both psychological and physical well-being among elderly individuals who remain in the workforce postretirement. These enhancements are particularly pronounced in areas such as autonomy and self-actualization, underscoring the profound impact of maintaining a purposeful role in the society beyond the traditional retirement age.

The positive correlation between postretirement work and enhanced QoL aligns with previous studies, such as those by Zhang et al. [99] and Hamzehnejadi et al. [100], which, respectively, explore the broader impacts of social engagement and wellness practices on individual well-being. Similar to Zhang et al.'s findings on the role of social media in enhancing educational outcomes through improved family bonding, our study suggests that continued work can enhance social bonds and community engagement among the elderly [99]. This integration not only enriches their personal lives but also contributes to societal health as a whole.

Furthermore, one of the critical gaps identified in our review is the limited focus on the intersection between postretirement work and QoL, especially in the context of Cyprus. The study fills this gap by providing empirical data that supports the development of policies aimed at integrating older adults more effectively into the workforce. By doing so, the study contributes to a more comprehensive understanding of aging, work, and well-being, highlighting the potential for policy innovations that could benefit both individuals and society at large.

Furthermore, this research challenges some existing narratives about retirement and economic independence, as discussed in the studies of Jaffar et al. [32] and Rehman et al. [101]. While these studies focus on the impact of socioeconomic factors on QoL, our findings suggest a nuanced view where psychological fulfillment from work significantly contributes to life satisfaction, independent of financial factors.

Moreover, working postretirement is associated with favorable effects on psychological self-confidence and physical energy, promoting a more optimistic outlook on life [75, 76]. Continuing postretirement work seems to be beneficial for the elderly since even those who have retired tend to regard work as contributing more favorably to psychological self-confidence and physical energy. Continuing work could therefore reinforce the concept of healthy aging [45], helping to increase the rate of mental and physical functioning, and strengthening the self-esteem and self-confidence of working pensioners who seem to be inspired by a more optimistic attitude towards life. This is also apparent from the perceptions of retired people who have ceased working because they tend to agree that continued work is beneficial both to physical functioning and to the psychological empowerment of retired people. This is likely to show that they would be interested, under appropriate circumstances, to work beyond retirement age because they want to remain active and not because they need the extra income that work offers [3].

Within this context of exploring the multifaceted benefits of sustained employment for the elderly, it is pertinent to consider the evolving nature of the working environment and the flexibility that is provided for the elderly. Some research studies further enrich this discussion by delving into diversity and inclusion within the workplace, specifically through the lens of teleworking. The investigation into the efficacy of teleworking arrangements among older employees underscores a critical finding: age significantly influences teleworkers' job performance. This insight suggests that teleworking not only complements traditional work modalities but also represents a valuable avenue for accommodating the unique needs of aging workers, offering them greater flexibility and the opportunity to maintain their contributions to the workforce without the physical demands of a conventional office environment. This revelation aligns with our understanding that continued engagement in work, whether through traditional or teleworking arrangements, plays a crucial role in enhancing the QoL among the elderly [102].

On the other hand, while the findings shed light on the benefits of postretirement employment, such as enhanced psychological and physical well-being, it is crucial to consider potential downsides. Continuous work engagement may infringe upon the leisure time and social interactions that are essential for a balanced life in retirement. Extended work commitments can lead to reduced opportunities for rest and personal pursuits, which may strain social relationships and decrease overall life satisfaction.

To mitigate these risks, the implementation of flexible work schedules is recommended to allow elderly workers to effectively balance employment with leisure and social activities. In addition, enhancing workplace support through ergonomic adjustments and mental health resources can help manage the physical and psychological demands placed on older employees. By adopting these strategies, employers can foster a more conducive environment that respects the unique needs of postretirement workers, ensuring that their continued employment contributes positively to their QoL without adversely affecting it.

Despite the evident challenges, including potential strain on physical health and possible limitations in engaging with leisure activities, the findings underscore the undeniable importance of continued employment postretirement. Specifically, it accentuates how such an engagement can remarkably enhance physical vitality, psychological well-being, and life satisfaction among the elderly population. The later finding is also supported in the study, as it transpired that the economic conditions and the financial security that pensioners have in Cyprus are not affected by their employment situation. Thus, this factor leading to empowerment and autonomy for the individual [51, 52], in the case of Cyprus seems to have little effect on the choice of retired people to continue to work or not. This is very interesting and seems to be consistent with some of the observations of the literature research by the authors in [30], accord-ng to which older people in low-income countries need to continue working simply because they cannot only live with a pension. In contrast, in higher-income countries where the pension system is more generous, older workers have a choice to withdraw from working life. In the case of Cyprus, as revealed by the study, the choice of prolonging working life is not affected by the need to increase income. Overall, retired people are financially self-sufficient and able to cope with their financial demands and obligations, with a greater degree of independence, of course, being shown to retired workers who still work.

Another factor that seems to affect the QoL of individuals is the social relationships that one can have with his friendly and family environment since they directly affect the psychological mood and general attitude of people towards life [49]. Considering, therefore, the possible effect of continuing work on the social relationships of individuals, since work is an environment in which the individual comes into contact with different people every day, unlike the home environment, it has been observed that work does not affect how pensioners experience their social relationships. Elderly people in Cyprus seem to satisfy their socialization needs by having their families close to them and other their relationships with their social environment, not seeking more social contacts.

Therefore, it appears that most of the pensioners in Cyprus positively assess their social contacts, regardless of whether they work or not. One possible explanation for this, as mentioned above, is the family structure in Cyprus, which follows the characteristics of an extended family in which grandparents and relatives reside at a relatively close distance from the nuclear family, interacting and offering support to each other. At the same time, people in Cyprus (as in Greece) tend to communicate more often with their social environment, visiting and calling each other more regularly than in some other countries.

Finally, an equally important factor for a good QoL for elderly people is their active participation in daily activities and processes through which they make sense in their lives but are also charged with (physical) energy [54, 59]. Examining this factor in relation to the working situation of pensioners, it was observed that continuing to work does not significantly affect the way pensioners in Cyprus utilize their free leisure time.

However, there was a tendency for those who work to slightly appreciate the way they exploit their free time more, judging that their continued work makes them more productive. The noneffect of work on the way pensioners seem to engage in leisure time activities correlates with the economic independence they all seem to enjoy, as participation in a variety of activities (beyond work) entails some financial independence. This is also demonstrated by the correlation of the five factors.

In addition, other factors apart from work may contribute to the QoL for the elderly. This was particularly revealed by the low R-squared values of the regression models. One such factor could be improvements in the urban infrastructure, particularly efficient and accessible public transportation, which emerges as a crucial factor in enhancing the QoL for the elderly [103–107]. Adequate and safe urban infrastructure would facilitate greater mobility and independence among the elderly population. This is particularly relevant in the context of Cyprus, where improvements in public transportation systems can significantly contribute to the autonomy, socialization, and overall well-being of the elderly. Such considerations are essential in creating age-friendly cities that support the autonomy and active participation of the elderly in daily life [108–113].

Despite the evident benefits, our cross-sectional study design limits our ability to definitively establish causal relationships. To strengthen causal inference in future research phases, we propose the integration of longitudinal study designs or more robust statistical methods such as mixed-effects models or instrumental variable analysis. Longitudinal designs would allow us to track changes in QoL over time, directly attributing variations to continued work engagement or retirement. This approach could clarify the directionality and magnitude of the observed effects, providing a more dynamic understanding of how postretirement employment impacts well-being.

In addition, employing more sophisticated statistical techniques could help control for unobserved heterogeneity and potential confounders not accounted for in a cross-sectional setup. For example, using an instrumental variable approach could mitigate bias by accounting for variables that influence both the likelihood of working postretirement and QoL, thus bolstering the validity of our causal claims. By adopting these methods, future studies could offer deeper insights into the causal pathways between postretirement activities and elderly well-being, enhancing the generalizability and applicability of the findings in similar contexts.

To sum-up, work after retirement was found to play a significant role in the elderly autonomy, self-actualization, and independence, enriching their lives with a purpose for living. On the other hand, the findings of this study prompt further exploration into the QoL of the elderly across different cultural contexts and the impact of various work and socioeconomic conditions on their well-being. For instance, longitudinal studies can provide deeper insights into the long-term effects of postretirement work, as well as comparative studies that include multiple countries or regions to better understand the cultural dimensions of aging and work.

### 5.1. Policy Recommendations and Implications for Management

Given the substantial impact of postretirement employment on the elderly, it is imperative to formulate policies that enhance the QoL. For instance, the government as well as corporations should consider introducing flexible employment policies that accommodate the unique needs of elderly workers, such as reduced working hours and adaptable job responsibilities. In addition, financial incentives could be provided to employers who make necessary adjustments to their workplaces to accommodate older employees. These adjustments could include ergonomic enhancements and the creation of part-time opportunities tailored to older adults.

Furthermore, lifelong learning initiatives are essential in ensuring that older adults remain competitive in the job market. These should include access to continuous education and training in new technologies and work-related skills. Employers should also integrate health and wellness programs specifically designed for older workers to help maintain their physical and mental health, which are crucial for their continued productivity and engagement in professional settings.

Furthermore, given the substantial impact of postretirement employment on elderly's well-being, it is imperative to formulate specific, actionable policy measures to facilitate the integration of older individuals into the workforce. One recommendation is to introduce tax incentives for employers who hire and retain older workers. Such incentives can offset potential costs associated with workplace adjustments and encourage businesses to value the contributions of elderly employees. In addition, government-funded retraining programs tailored to older adults can help them acquire new skills and stay competitive in the job market. These programs should focus on digital literacy, emerging technologies, and other relevant skills to ensure that the elderly can adapt to modern work environments. Furthermore, policies should promote flexible work schedules and part-time opportunities, allowing older workers to balance employment with personal and social activities. By implementing these measures, policymakers can support the continued engagement of older individuals in the workforce, enhancing their QoL and contributing to broader societal well-being.

The implications of encouraging postretirement employment are extensive and multifaceted. Economically, supporting elderly employment can mitigate the negative impacts of an aging population by maintaining a larger, more experienced workforce, which is crucial for sustaining economic growth. Healthier and actively working elderly populations can lead to reduced healthcare costs, benefiting public health systems and the economy at large. From a social perspective, continued employment can help prevent social isolation among the elderly, contributing to better mental health and more integrated communities.

Thus, a significant opportunity arises for policymakers, to develop strategies that not only support aging workers but also contribute to building a more inclusive society. Public awareness campaigns are also recommended to shift societal attitudes towards older workers and highlight the productivity and value added by elderly employees across various sectors.

Implementing these recommendations will provide policymakers with a robust strategic toolkit to enhance the well-being of the elderly, thereby fostering a prosperous society that values and supports its aging population. This approach will particularly lead to a more dynamic and inclusive workforce, benefiting individuals and the broader economic and social fabric of Cyprus.

## 6. Conclusion

This paper has illuminated the significant, positive impact of postretirement organizational work on the QoL based on a survey study administered to elderly individuals in Cyprus. Specifically through the statistical analysis of the collected survey data, it has been revealed that there are notable enhancements in autonomy, self-actualization, physical well-being, and psychological health as a result of continued professional engagement beyond the traditional retirement age. Contrary to prevailing assumptions that depict retirement as a period of inevitable decline, the findings of the present study suggest that ongoing work can substantially contribute to a more active and fulfilling elderly life. Furthermore, research findings also suggest that postretirement work is not necessarily carried out for financial independence. The nuanced results suggest that the benefits of continued work are primarily psychological in nature rather than economic. Such insights challenge the multifaceted nature of work and its broader impacts beyond mere financial sustenance.

The global relevance of these findings is also important. While they are immediately pertinent to the Cypriot context, they also resonate with broader international discussions about aging populations and workforce participation. Thereby, the present study extends the body of knowledge by demonstrating the potential for postretirement work to enrich the QoL, laying a foundation for further research into similar interventions across different cultural and economic landscapes.

As a result, significant implications arise for businesses, policymakers, and social planners. Such implications advocate for policies that support a variety of work options for the elderly to capture the evolving discourse on aging, work, and well-being. Such policies can help integrate the aging population more effectively into the workforce, thus not only enhancing individual lives but also leveraging their potential to contribute to societal welfare.

Summing-up, the above findings enhance our understanding of the multifaceted impacts of work on elderly well-being and serve as a valuable reference for shaping policies aimed at enhancing the quality of elderly life. It is recommended that future studies continue to explore this promising area of research, potentially examining longitudinal impacts and the effects of various types of work to provide even deeper insights into the complex dynamics involved.

## Figures and Tables

**Table 1:** Demographics.

Demographic	*N*	Percent	Mean	SD
Gender				
Male	75	62.00%		
Female	46	38.00%		
Age			70.50	4.48
Education				
Primary	44	36.40%		
Lower secondary	23	19.00%		
Upper secondary	35	28.90%		
1st cycle tertiary	15	12.40%		
2nd/3rd cycle tertiary	4	3.30%		
Work status				
I do not work	81	66.90%		
I do work	40	33.10%		

**Table 2:** Comparing mean scores between the elderly who work after retirement and the elderly who do not work.

No.	Question	Not working		Working		All		*t*-stat	*p* value
		*N* = 81		*N* = 40		*N* = 121			
		m	SD	m	SD	m	SD		
1.	I generally enjoy my life as a whole	3.6	0.9	3.9	0.6	3.7	0.8	−1.66	0.099
2.	I look forward to the things that come in the future	3.3	1.0	3.4	0.8	3.3	0.3	−0.705	0.482
3.	Most of the time I'm happy	3.7	0.9	3.6	0.7	3.6	0.6	0.488	0.626
4.	Aging takes me down	2.4	0.9	2.0	0.7	2.3	0.9	2.104	0.037
5.	I have a lot of physical energy	3.2	1.0	3.9	0.7	3.4	1.0	−3.917	< 0.001
6.	Physical fatigue affected my choice to continue or not to work after I got a pension	2.8	1.2	3.4	1.4	3.0	1.3	−2349	0.020
7.	My health status prevents me to take care of myself and my home	2.4	1.3	2.1	1.0	2.3	1.2	1.176	0.242
8.	I am healthy enough to do whatever I want	3.5	1.2	3.8	0.7	3.6	1.1	−1.512	0.130
9.	Family, friends and neighbors help me if necessary	3.8	0.9	3.9	0.7	3.8	0.9	−0.285	0.776
10.	I would like to have more social contacts	3.5	0.9	3.1	0.9	3.3	0.9	2.479	0.015
11.	I have enough contacts with different kinds of people	3.4	1.0	3.8	0.7	3.5	0.9	−1.947	0.054
12.	I'm close to my children	4.2	0.8	4.3	0.6	4.2	0.8	−0.605	0.546
13.	I feel I have more independence as a result of working	3.4	1.2	4.1	0.9	3.6	1.1	−3.699	< 0.001
14.	The cost of living in relation to my income limits my lifestyle activities	3.5	1.1	3.4	1.0	3.5	1.0	0.704	0.483
15.	I feel I have more control over my future if I continue to work	3.2	1.1	4.0	0.9	3.5	1.1	−3.983	< 0.001
16.	I have more freedom to decide for myself when I work	3.3	1.1	4.2	0.9	3.6	1.1	−4.022	< 0.001
17.	I feel more satisfied with my life when I work	3.4	1.1	4.4	0.7	3.7	1.1	−4.86	< 0.001
18.	I feel lucky compared to most of my friends	3.7	0.9	3.7	0.8	3.7	0.9	0.24	0.811
19.	I feel more productive if I continue to work	3.5	1.2	4.2	0.7	3.8	1.1	−3.252	0.001
20.	I find continuously new things to do	3.4	1.0	3.5	0.7	3.4	0.9	−0.472	0.638
21.	I am satisfied with the way I use my time	3.6	1.1	3.8	0.6	3.7	0.9	−1.068	0.280
22.	I feel more recognized when I work	3.5	1.1	3.8	0.8	3.6	1.0	−1.252	0.213
23.	I have enough money to pay all my bills	3.4	1.0	3.7	0.8	3.5	1.0	−1.358	0.177
24.	I have enough money to pay for any changes needed at home at any time	3.0	1.1	3.0	0.8	3.0	1.0	−0.193	0.847
25.	I have the ability to buy whatever I need at any time	2.8	1.1	3.1	0.9	2.9	1.0	−1.399	0.164
26.	I have the financial ability to do all the things I enjoy	2.9	1.2	3.4	0.9	3.0	1.1	−2.349	0.020
27.	I can better participate in activities and enjoy if I do not work	3.3	1.1	3.2	1.1	3.2	1.1	0.336	0.737
28.	I remain active as I was before retirement	3.3	1.1	3.8	0.7	3.5	1.0	−2.532	0.013
29.	To continue working gives me a purpose in my life	3.5	1.1	4.0	0.9	3.6	1.0	−2.711	0.008
30.	I have a busy day if I continue to work	3.5	1.1	4.0	0.8	3.6	1.0	−2.964	0.004
31.	I do not find time to participate in things that please me because of work	3.3	1.0	2.4	1.0	3.0	1.1	4.991	< 0.001

**Table 3:** Factor analysis.

Statement	Autonomy and self-actualization	Financial independence	Physical well-being	Socialization	Leisure time	% of explained statements⁣^∗^
I have more freedom to take my own decisions if I am working	0.858	0.005	−0.108	−0.127	−0.042	0.766
I have more control over my future if I keep working	0.857	−0.108	−0.09	−0.023	−0.058	0.758
I feel more satisfied if I am working	0.805	−0.07	0.119	0.085	−0.05	0.676
I feel more productive if I am working	0.799	−0.015	0.084	0.228	0.134	0.715
My contributions are more recognized if I am working	0.789	0.008	−0.045	0.02	0.033	0.626
Working gives my life a purpose	0.785	−0.127	0.072	0.101	0.158	0.673
I have a more fulfilling day if I keep working	0.782	−0.126	0.079	0.001	0.186	0.668
I have more independence if I keep working	0.768	−0.061	0.066	−0.147	−0.058	0.624
I have enough money to pay for any work needed for my house at any given time	−0.089	0.892	0.046	−0.014	0.052	0.808
I have enough money to buy whatever I need at any given time	−0.166	0.852	0.149	0.046	0.127	0.794
I have enough money to pay for all my bills	−0.035	0.815	0.143	0.073	−0.139	0.71
I have enough money to do all the things that I enjoy doing	−0.063	0.814	0.219	0.091	0.239	0.78
I have a lot of physical energy	0.083	0.242	0.824	−0.079	−0.036	0.752
I am sufficiently healthy to do whatever I want to do	0.197	0.125	0.807	0.052	0.202	0.75
I take part in activities I did before retirement	−0.149	0.136	0.76	0.208	0.131	0.679
My family, friends, and neighbors will help me if needed	−0.047	0.159	0.11	0.834	−0.14	0.755
My children are near me which is very important	0.106	−0.01	0.026	0.758	0.227	0.638
I am excited for the things that are coming now that I have retired	0.131	0.122	−0.003	0.035	0.802	0.677
Now, I am continually finding new things to do	−0.012	0.059	0.384	0.056	0.699	0.643
Percentage of explanation of total dispersion	28.1%	16.0%	11.7%	7.6%	7.6%	
Self-values	5.53	3.81	1.72	1.32	1.11	

*Note:* Extraction method: principal component analysis. Rotation method: varimax with Kaiser normalization.

⁣^∗^Communalities: The percentage of explanation of dispersion of each question that is explained by the five factor model.

**Table 4:** Regression analysis of the factors influencing work beyond retirement.

	Autonomy and self-actualization			Financial independence			Physical well-being			Socialization			Leisure time		
	*B*	Std. error	*p* value	*B*	Std. error	*p* value	*B*	Std. error	*p* value	*B*	Std. Error	*p* value	B	Std. error	*p* value
(Constant) Work	3.418	0.091	< 0.001	3.015	0.098	< 0.001	3.333	0.091	< 0.001	3.66	0.072	< 0.001	3.333	0.082	< 0.001
	0.669	0.158	< 0.001	0.266	0.17	0.121	0.5	0.158	0.002	−0.198	0.126	0.119	0.104	0.143	0.468
	*F*(1.119) = 17.840, *p* < 0.00)			*F*(1.119) = 2.436, *p*=0.121)			*F*(1.119) = 9.958, *p*=0.002)			*F*(1.119) = 2.468, *p*=0.119)			*F*(1,119) = 0.531, *p*=0.468)		
	Adj *R*^2^ = 0.123			Adj R2 = 0.012			Adj *R*^2^ = 0.069			Adj *R*^2^ = 0.012			Adj *R*^2^ = 0.004		

**Table 5:** Average level, regularity, and credibility of factors.

	No. of forms	Mean	S.D.	K–S test (z)	*p*	Cronbach's' alpha
Autonomy and self-Actualization	8	3.6	0.9	1234	0.095	0.925
Financial independence	4	3.1	0.9	0.99	0.281	0.888
Physical well-being	3	3.5	0.8	1544	0.017	0.783
Socialization	2	3.6	0.7	1952	0.001	0.519
Leisure time	2	3.4	0.7	2054	< 0.001	0.489

*Note:* K–S, Kolmogorov–Smirnov test.

**Table 6:** Spearman factors' correlations.

	Autonomy and self-actualization	Financial independence	Physical well-being	Socialization	Leisure time
Autonomy and self-Actualization	—				
Financial independence	−0.163	—			
Physical well-being	0.07	0.352⁣^∗∗^	—		
Socialization	−0.1	0.115	−0.033	—	
Leisure time	0.115	0.209⁣^∗^	0.354⁣^∗∗^	0.048	—

⁣^∗^Correlation is significant at the 0.05 level (2-tailed)

⁣^∗∗^Correlation is significant at the 0.01 level (2-tailed).

## Data Availability

Data are available from the last author upon request (Prof Andreas Efstathiades, a.efstathiades@euc.ac.cy).
